# Prevalence and Correlates of Water, Sanitation, and Hygiene (WASH) and Spatial Distribution of Unimproved WASH in Nepal

**DOI:** 10.3390/ijerph19063507

**Published:** 2022-03-16

**Authors:** Shalik Ram Dhital, Catherine Chojenta, Tiffany-Jane Evans, Tri Dev Acharya, Deborah Loxton

**Affiliations:** 1 Centre for Women’s Health Research, School of Medicine and Public Health, University of Newcastle, Callaghan, NSW 2308, Australia; catherine.chojenta@newcastle.edu.au (C.C.); deborah.loxton@newcastle.edu.au (D.L.); 2 National Health Education, Information and Communication Centre, Ministry of Health, and Population, Kathmandu 44600, Nepal; 3 Hunter Medical Research Institute, New Lambton Heights, NSW 2308, Australia; tiffany_e16@hotmail.com; 4 Institute of Transportation Studies, University of California Davis, Davis, CA 95616, USA

**Keywords:** household, improved, unimproved, WASH, spatial distribution, Nepal

## Abstract

This study aims to estimate the prevalence and correlation of household levels of water, sanitation, and hygiene (WASH), including the identification of areas where WASH facilities are unimproved in Nepal. The study population was 11,040 household heads, using the data collected in the Nepal Demographic and Health Survey 2016. Logistic regression analysis was performed and crude odds ratios (OR) with 95% confidence intervals (CI) using a 0.05 significance level are presented. Getis–Ord Gi* statistics were used to identify the hot and cold spot areas of unimproved WASH. GPS locations of WASH points were used for spatial analysis. Approximately 95% of households had an improved water source, 84% had improved sanitation facilities, 81% had a fixed place for handwashing, and 47% had soap and water. Education, wealth, and ecology were significantly associated with WASH. The people from the hills were less likely to have an improved water source (OR = 0.32; 95% CI: 0.16–0.64) than those from the plain. Households with a poor wealth index had 78% lower odds of having an improved water source compared to households with a rich wealth index. Respondents from Madhes Province had lower odds (OR = 0.15; 95% CI: 0.08–0.28) and Gandaki Pradesh had the highest odds (OR = 2.92; 95% CI: 1.52–5.61) of having improved sanitation facilities compared to Province 1. Respondents aged 35–44 years had higher odds (OR = 1.16; 95% CI: 1.04–1.29) of having soap and water available compared to those aged 45 years and older. Education and geographical disparities were the factors associated with having reduced access to WASH. These findings suggest the need to focus on advocacy, services, and policy approaches.

## 1. Introduction

Globally, approximately 82% of people have access to an improved water source (piped water (piped into dwelling, piped to yard or plot, piped to neighbour), a public tap (standpipe), a tube-well (borehole), a protected dug well, a protected spring (natural source), rainwater, and bottled water), 78% of people have access to improved sanitation, also considered as “sanitary toilet facilities” for this study (flush and pour-flush toilets to the piped sewer system; flush to septic tanks; flush to pit latrines; ventilated improved pit latrines; pit latrines with slabs; and composting toilets without sharing (and/or with sharing with other households)), and 70% of people have access to handwashing facilities with soap in the home in 2020 [1]. However, 18% of people have a lack of access to an improved water source, including limited water sources and surface water. About 21% of people have a lack of access to improved sanitation or sanitary toilet facilities, including limited facilities and open defecation, and 30% of people have a lack of access to basic handwashing facilities. The prevalence of access to water, sanitation, and hygiene (WASH) varies by country, region, geography, and rurality. For this study, the definition of adequate water, sanitation, and hygiene is access to “an improved water source, improved sanitation or a sanitary toilet, and handwashing with soap and water in a fixed place” [2].

Unimproved WASH is a risk factor for communicable diseases [3,4,5], and these are associated with a 50% increased risk of diarrhoea globally [6]. The promotion of WASH is important for human health, wellbeing, and overall development [7,8]. Sustainable Development Goal (SDG) 6, 2016–2030, and the Nepal Health Sector Strategy Implementation Plan (NHSS–IP), 2016–2021, give priority to WASH in Nepal. However, WASH is often forgotten during health planning in developing countries, including Nepal [9]. The current global pandemic of COVID-19 highlighted the importance of WASH, including handwashing with soap, as a pivotal health issue [10].

Providing facilities for adequate WASH in a sustainable way is a cost-effective measure for the prevention and control of communicable diseases [11,12]. Approximately 10% of the total global burden of diseases can be prevented with improved WASH [13]. Previous studies showed that the lack of adequate, improved WASH practices is due to lack of knowledge [14], poverty [15], lack of political commitment and political instability, lack of coordination and poor management of available, WASH-related resources and infrastructure [12], gender bias [16], geographic constraints, and socio-cultural factors [12,17].

Existing studies that address socio-demographic and contextual factors relating to WASH in Nepal are limited. The few studies that examined WASH at the household level had small sample sizes and focused on the regional community level and, thus, may lack generalizability [18]. A study carried out by Wang et al., in 2019, examined access to water and sanitation; however, handwashing was not assessed. Furthermore, Wang et al.’s results were inconsistent compared to the Nepal Demographic and Health Survey (NDHS) report [19], which provides a nationally representative view of WASH in Nepal. A scoping review by Wali et al., in 2020, found that women are vulnerable to lack of access to WASH facilities in Nepal [20]. The utilization of a database, such as NDHS, in research, is important for providing a clear direction for effective planning and development of interventions and for examining the geographic distribution of WASH. To date, there are no studies conducted on WASH facilities using NDHS data that include spatial analysis to identify the areas with a high prevalence of unimproved WASH facilities in Nepal.

This study aimed to estimate the prevalence and correlation of household levels of water, sanitation, and hygiene (WASH), including identification of areas where WASH facilities are unimproved in Nepal. This study was conducted using the NDHS 2016 dataset, and the results are presented based on such data. The published scientific articles and reports based on data analysis available after this study are discussed only in the discussion chapter.

## 2. Materials and Methods

### 2.1. Study Design and Data Source

The NDHS 2016, a nationally representative household survey, was used to conduct this study [21]. The lead role in the NDHS 2016 was taken by the Ministry of Health and Population, Nepal, with the financial assistance of the United States Agency for International Development (USAID) and technical support from the Inner City Fund (ICF) International (a global consulting and technology service company). The main implementation partner of this survey was New ERA, a non-government and non-profit research organization under the supervision of the Ministry of Health and Population, Nepal. The survey was carried out from 19 June 2016 to 31 January 2017. 

The NDHS 2016 survey used an updated version of the sampling frame of the National Population and Housing Census 2014. The update was needed because the rural and urban classification changed at the ward level after the restructuring of the nation in 2015. Some new places were declared as municipalities, and some were re-framed. These changes were divided into seven provinces. The seven provinces were further stratified into 14 strata (rural and urban areas) and comprised 77 districts after splitting Rukum District and Nawalparasi District into two. The rural and urban areas were further divided into wards, the smallest administrative block (primary sampling unit, PSU); however, urban areas were further divided into enumeration areas (EA) due to having more households than rural areas.

### 2.2. Sample Selection

A total of 11,490 households were selected using a two-stage stratified cluster sampling technique in rural areas and a three-stage stratified cluster sampling technique in urban areas. In rural areas, in the first stage of sampling, PSUs were selected by probability proportional to size, followed by systematic selection of households from individual PSUs in the second stage of sampling, while, in urban areas, in the first stage, PSUs were chosen by probability proportional to size. In the second stage, EAs were randomly selected from PSUs, followed by a systematic selection of households applied in the final stage of sampling.

**Figure 1:**
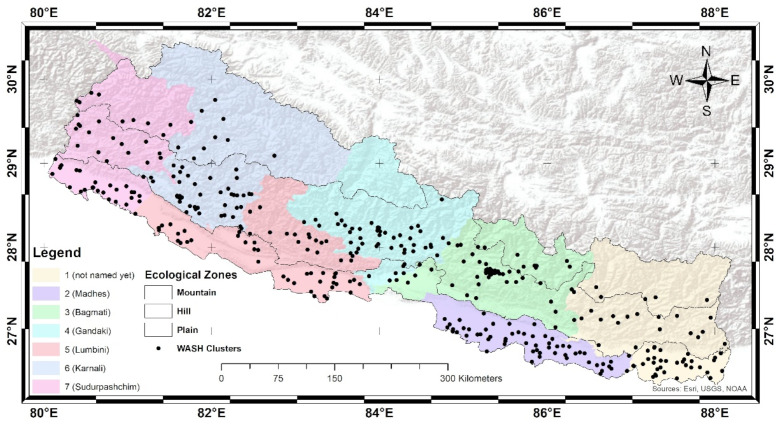
The study area, Nepal, with the province and ecological zones. Each dot represents WASH clusters (ward as a primary sampling unit; *n* = 363).

A total of 383 wards (199 rural and 184 urban, Figure 1), representing mountains, hills, and plains zones, were selected, and the interview was applied to 11,040 residential households head (6978 rural and 4062 urban). A fixed number of 30 households in each ward was selected, with an equal selection from the household listing. All analyses used the sampling weights calculated for each interviewed household. A questionnaire was used to collect information from household heads. In this study, the household record file (HR dataset) was used for analysis.

### 2.3. Predictors

Socio-demographic characteristics of respondents were selected because these variables are found to be factors affecting WASH services [22,23,24]. These predictors (Table 1) were selected based on the criteria of each variable, which were evaluated individually with outcome variables by applying bivariate analysis. This model was unadjusted to determine the crude effect size of variables [25]. A chi-squared test was performed to determine whether there was a significant difference between the predicted frequencies and the observed frequencies in each of the categories.

### 2.4. Outcome Variables

The outcome variables for this study included access to an improved water source, improved sanitation, a fixed place for handwashing, and soap and water for washing at the household level. In the NDHS 2016, the WASH-related information was taken from both household interviews and by observation by the data collector. Dichotomous variables for each WASH outcome were derived from the collected data. Access to an improved water source included: if the household had piped water (piped into dwelling, piped to yard or plot, piped to neighbour), a public tap (standpipe), a tube-well (borehole), a protected dug well, a protected spring (natural source), rainwater, and bottled water. Improved sanitation was defined as those that flush or pour to a piped sewer system or septic tank, composting toilets, and those that flush or pour to a pit latrine ventilated improved pit latrine or pit latrines with slabs. The type of toilet facilities was collected through observation. A fixed place for handwashing was defined as a dedicated, convenient location where water and soap were provided [26]. Soap and water were categorized as being available when both were provided in a handwashing place. The information about handwashing facilities was collected through observation during data collection [21].

Water service was considered as safely managed, basic, or limited; the sanitation facilities were categorized according to human excreta disposal types, such as sewer connection, onsite sanitation, unimproved, and no facility, and the handwashing facilities were categorized into basic or limited during the Millennium Development Goals (MDGs) and SDGs monitoring, as per Joint Monitoring Programme (JMP) criteria. In this study, we considered improved and unimproved water, sanitation, and hygiene as they are considered as dichotomous variables for each outcome. Additionally, the data obtained from NDHS 2016 for this study were not available for the safely managed, basic, and limited services. Therefore, we mentioned improved and unimproved WASH facilities throughout the document according to the classification of DHS-7 guidelines.

### 2.5. Statistical Analysis

The STATA 15 was used to analyze data for this study [27]. A univariate analysis was conducted of the socio-demographic and WASH characteristics of respondents. The respondents’ characteristics were presented in the form of weighted frequencies (*n*) and percentages weighted for sampling distribution (%). The Rao–Scott F-adjusted chi-squared test was performed to determine whether there was a significant difference between the predicted frequencies and the observed frequencies in each of the categories [28]. This test gives more accurate results than other traditional methods. Weighted samples increase the probability of selection of samples in each region and province. A bivariate analysis (cross-tabulation) between dependent variables (for example, improved source of water, improved sanitation, handwashing with soap, and a fixed place for handwashing) and independent covariate or explanatory variables was performed to estimate crude odds ratios (OR) with 95% confidence intervals (CI) using a 0.05 significance level.

### 2.6. Spatial Analysis

The spatial mapping was conducted using ArcGIS, 10.6.1 version (Environmental Systems Research Institute, Redlands, CA, USA). The administrative boundaries of Nepal were used to link data and as a base map for the analysis. Nepal lies between latitudes 26°22′ N to 30°27′ N and longitudes 80°04′ E to 88°12′ E [29]. The universal transverse Mercator zone between 44 degrees and 45 degrees north was used as a projection with 0.9996 as the scale factor for the central meridian.

Proportionally distributed WASH-related data obtained from NDHS 2016 dataset were joined with each cluster to the corresponding, geospatial location (ward) or survey cluster values. The values of the NDHS 2016 data were linked with the coordinate, and the mapping clusters were estimated using hot-spots analysis (Getis–Ord Gi*) via Geoda software (Center for Spatial Data Science, Chicago, IL, USA).

The positive and negative Moran’s autocorrelation was applied to determine the high value and low value based on the z-score results [30]. The statistically significant autocorrelation was estimated based on z-scores with a *p*-value with 95% CI. The positive autocorrelation (yielding a positive z-score) indicates similar values clustered together on a map corresponding to high rates surrounded by nearby high rates or low rates surrounded by nearby low rates. The negative autocorrelation (yielding negative z-score) indicates dissimilar values clustered together on a map corresponding to high rates surrounded by nearby low rates or low rates surrounded by nearby high rates. The high and low clustering patterns are presented. The critical value of a positive z-score (>2.58 at 0.01 significance level, 1.96 to 2.58 at 0.05 significance level, and 1.65 to 1.96 at 0.10 significance level) value indicates a high value for the unimproved WASH, while if the critical value of the z-score is negative (<−2.58 at 0.01 significance level, −1.96 to −2.58 at 0.05 significance level, and −1.65 to −1.96 at 0.10 significance level), the clustering is smaller than expected, which indicates that the low values are clustered in the study. If the z-score is calculated between −1.65 and 1.65, it indicates that there is no significant relationship.

### 2.7. Ethical Consideration

Ethical approval for this study was obtained from the Human Research Ethics Committee of the University of Newcastle. The Inner City Fund’s Institutional Review Board and the Demographic and Health Survey Program in Maryland, USA, provided approval for the use of the NDHS 2016 data for this study. The Ethical Review Board of the Nepal Health Research Council, Kathmandu, provided ethical approval before the NDHS in 2016. All study respondents were appropriately informed about what was involved in participating in the survey and gave written consent before the interview and observation. Respondents were assured their personal details would remain confidential.

## 3. Results

### 3.1. Descriptive Statistics

Table 2 summarizes the socio-demographic and WASH characteristics of the respondents. Of the 11,040 household heads, the majority were aged 45 years or older (*n* = 5631, 50.8%). Nearly one-third (*n* = 3459, 31.3%) of households were headed by women. Approximately 39% of the respondents had no formal education, and the literacy rate was 61%. About 22.5% of the respondents had a primary level of education, 26.7% had a secondary level of education, and a minority (11.5%) had a SLC from high school or a higher level of education. Approximately 86.0% of household heads were married, 10.9% were widowed and divorced, and only 3.1% were unmarried.

Regarding the rural/urban setting, the majority of the interviewed households lived in a rural area (*n* = 6019, 54.5%). Respondents were from the plains (46.4%), hills (46.5%), and mountains (7.1%) regions. The provincial representation of respondents was unequal. The highest proportion of respondents lived in Bagmati Pradesh (*n* = 2521; 22.9%). The percentages of people in the poor and rich categories on the wealth index were similar across the households (40.4% and 40.9%, respectively), while the percentage in the middle category of the wealth index was much lower at 18.7%.

The prevalence rates of having an improved water source and improved sanitation were 95.5% and 83.8%, respectively. Of the households with a handwashing place (which was 80.9%), approximately 46.9% had both soap and water available. The majority (*n* = 10,476, 94.9%) of households had a drinking water source less than 30 min walk from their house, and only 5.1% of households spent more than 30 min walking to collect water.

### 3.2. Correlation of WASH Factors

Table 3 shows household heads who had obtained a high school or higher level of education had a higher chance of having an improved water source relative to those with no education. Households that had to travel more than 30 min to their water source, people from the hills communities, households in Karnali Pradesh, and the poor category of household wealth had lower rates of access to an improved water source. Age, sex, marital status, number of family members, and place of residence were not significantly correlated to water source status.

Table 3 also shows the association between possible predictors and having improved sanitation in the household. Having a high school or higher education level, never-married household heads, and those living in urban areas and Gandaki, Karnali, and Sudurpashchim Pradesh were factors highly related to improved sanitation. However, in Madhes Province, household wealth index groups from the poor and middle categories had lower rates of improved sanitation (Table 3). The age, sex, and distance to a water source of the household head were not significantly related to having improved sanitation. Age, education, marital status, number of family members, distance to a water source, place of residence, ecology, province, and wealth index were significantly related to having soap, water, and a fixed handwashing place (Table 3).

### 3.3. Visualization of WASH Hot and Cold Spots

**Figure 2:**
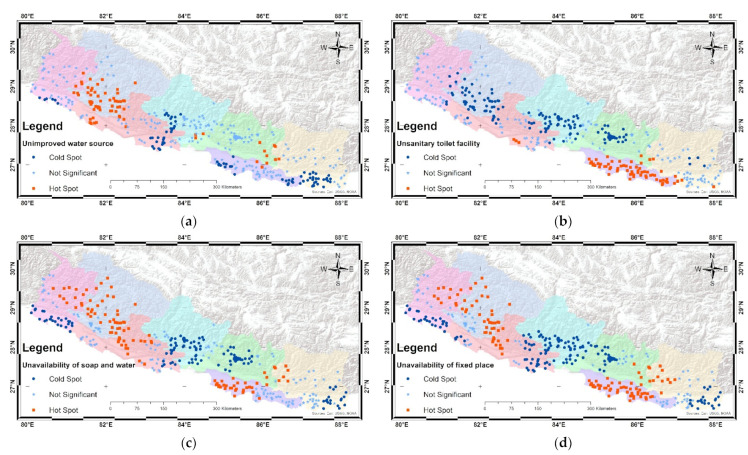
Mapping cluster in Nepal: (**a**) unimproved water source; (**b**) unimproved sanitation; (**c**) unavailability of soap and water; and (**d**) unavailability of a fixed place.

The distribution of unimproved water sources, unimproved sanitation, unavailability of soap and water, and absence of a fixed place for handwashing are visualized in Figure 2a–d. This indicates the spatial variation of unimproved WASH at the cluster level. The hot spot indicates positive autocorrelation and the cold spots negative autocorrelation. The spatial analysis at the cluster level indicates that statistically significant high values (hot spots) of unimproved water sources were found in parts of the far- and mid-western hills (Karnali and Sudurpashchim Pradesh) of the country, whereas statistically significant low values (cold spots) of unimproved water sources were found in the central hills and plains regions (Gandaki and Lumbini Pradesh) and south-east plains (Province 1 and Madhes Province) of the country.

The spatial analysis at the cluster level indicates that statistically significant high values (hot spots) of unimproved sanitation were found in parts of the southern plains of the country (Madhes Province), whereas statistically significant low values (cold spots) of unimproved sanitation were found in parts of the central and western hills (Bagmati, Lumbini, and Karnali Pradesh) of the country. The hot values (hot spots) are, therefore, those states where households are at high risk of communicable diseases.

The spatial analysis at the cluster level indicates that statistically significant high values (hot spots) of unavailability of soap and water and also a fixed place for handwashing were found in the south plains (Madhes Province) and mid- and far-western hills and mountains (Karnali and Sudurpashchim Pradesh) of the country, whereas statistically significant low values (cold spots) of unavailability of soap and water and also a fixed place for handwashing were found in the eastern plains (Province 1), as well as in parts of the central and western hills (Bagmati, Gandaki, and Lumbini Pradesh) of the country.

## 4. Discussion

This study aimed to estimate the prevalence and correlation of household levels of WASH, including the identification of areas where WASH facilities are unimproved in Nepal. There are a few studies related to WASH that have been performed in Nepal [1,11,16,17,31]. However, there is a lack of studies measuring each WASH component, such as access to a source of water, toilet facilities, availability of soap, water, and fixed places for handwashing, in Nepal, including spatial analysis. This study highlights the need to provide the current status of each WASH component and create an enabling environment for sustainable WASH facilities for policymakers and WASH implementers. The United Nations’ SDG 6 for 2016–2030 focuses on achieving adequate and equitable universal access to sanitation and hygiene by 2030 [32,33].

At the household level, the prevalence of having an improved water source was 95.5%, improved sanitation was 83.8%, a fixed place for handwashing was 80.9%, and availability of both soap and water was 46.9%. Education, number of family members in the home, ecological zones, provinces, and household wealth index were statistically significantly related to household WASH in Nepal. Marital status and rural/urban setting were also significant predictors of a household having improved sanitation, a fixed place for handwashing, and availability of soap and water. The results show variation in the coverage of WASH which might be due to several contributing factors, including geographical discrepancies, number of family members in the home, level of education, and economic status. The spatial distribution of WASH components varied by province. This study shows the disparities in the WASH coverage by ecological zones and provinces. Karnali Pradesh was a hot spot of unimproved water sources compared to other provinces; Madhes Pradesh was a high hot spot of unimproved sanitation; and the absence of handwashing facilities was a high hot spot in Madhes Pradesh, Karnali Pradesh, and the plains part of Sudurpashchim Pradesh. The household distribution of WASH was determined according to cluster-level WASH facilities. It can be assumed that, if there is a sound WASH situation in the cluster distribution, then the WASH supply at the household level can also be considered improved.

The rate of having an improved water source in Nepal was higher than the global figure of 69% [34]. In previous years, in 2006 [35] and 2011 [36], the rates of improved water sources in Nepal were 28% and 38%, respectively, which were lower rates than this study. A study conducted in 2018, in Makwanpur District, showed that the prevalence of improved water sources was slightly lower than this study [18]. The high level of improved water sources in Nepal is most likely due to Nepal being a rich country in terms of water, as many rivers originate in the Himalayan Mountains [37]. Madhes, Gandaki, and Lumbini Pradesh had significantly higher rates of improved water sources compared to Bagmati and Karnali Pradesh. This may be due to Madhes Pradesh and Lumbini Pradesh being in the plains region and most households using groundwater which is easily available through a tube well. Gandaki Pradesh is an area with readily available pipe water and high rainfall. In another way, Karnali Pradesh (in the western hills and mountain regions) and Bagmati Pradesh (mostly the national capital) have fewer improved water sources because the capital city of Nepal is polluted by sullage and sewage, and they are mixed with water in the connection pipes or reservoirs of water, and the water becomes contaminated. Karnali Pradesh is a dry region because rainfall water sources are limited and, ultimately, people directly drink water from the river without any treatment.

Globally, in 2010, 29% of rural residents and 80% of urban residents had access to an improved water source. In contrast, in the present study, the proportional distribution of improved water sources in rural households was 54.6%, and, in urban households, it was 45.4%. The lower rates of urban, improved water sources compared with the global figure might be due to Nepal’s sullage and sewage disposal system being unimproved, and, when sullage and sewage enter a water source, this leads to water contamination [38].

The gap between rural and urban access to an improved water source in Nepal is due to several reasons. For instance, Nepal’s rural areas have many springs which are sources of improved water [39,40]. In rural areas there is a low population density compared with urban areas, meaning that rural areas are less exposed to water contamination. In a study conducted in Uttar Pradesh, rural India, in 2013, of the 1088 households, fewer than half had access to an improved water source [41], whereas, in the current study, more than double households had access to an improved water source. This disparity is seen because the India-based study covered a small sample, and Uttar Pradesh is one of the poorer, rural regions of India [42]. In 2012, about four in five people in Bangladesh had access to an improved water source [43]. The present study findings were comparable with the Bangladesh-based report. 

Improved water can also become contaminated during transportation and handling [44]. The improved water sources in Nepal could become polluted due to sewage where, mostly, *Escherichia coli* are present, and also agricultural residues, industrial effluents, and chemical substances [45]. Similar studies were carried out in 15 countries in Sub-Saharan Africa (SSA), where improved water sources were estimated using a DHS dataset. The average percentage of households with an improved water source was 77% (92% in urban areas, 62% in rural areas) in those countries in SSA [46], indicating that the present results in Nepal were higher. This differential rate also indicates that in some African countries (for example, Namibia), almost 90% of people have access to an improved water source, whereas, in other African countries (for example, Madagascar), only 50% or less have access to an improved water source. This present study’s results differed from the pooled average result of the Africa-based study [46]. Similarly, these multi-country-based differences might result from the varying periods of measurement in the different countries. Another study found that 92% of households in Nepal had access to an improved water source; the highest coverage was in the Bara district (100%), and the lowest access was in the Doti district (42%) [47]. Together with the current results, these findings highlight the large variation in access across the country and the need to assess drivers of access at the local level.

The benefits of using sanitary toilets for human excreta disposal as a cost-effective health-promotion strategy are well-known. Evidence suggests that improved sanitation reduces the incidence of diarrhoea by 50% [6,48]. The current research revealed that there were, overall, improved sanitation conditions, with a slight difference between mountains (92%), hills (94%), and plains (72%) regions, with an aggregate prevalence rate of 84% across Nepal. This means that 16% of households do not have improved sanitation facilities. The households from the plains region (Madhes Province) had the lowest rate of available improved sanitation. This may be due to overcrowding, urbanization, lack of knowledge about the importance of improved sanitation, poverty, and socio-cultural influences. It is evident that the government of Nepal has had a massive push to achieve country-wide ODF and, indeed, declared ODF in Nepal, but less priority has been given to sanitation and hygiene at each operational level for its maintenance and sustainable function [49,50]. The present study results demonstrated there was a higher availability of improved sanitation in Nepal than the baseline of the SDG rate, 67.7% [33,51] The overall figure was similar to the pooled result from 13 African countries of 75% of people with improved sanitation [46]. The result of this present study on improved sanitation coverage in Nepal was similar to the status in India.

A few distinctions between this present study and previous studies were found due to period differences, different approaches to public health, and context-specific issues. A study completed in 2018 showed that approximately 96% of people had access to safely managed human excreta in rural Bangladesh [50], a better result than in the present study. This is likely due to Bangladesh’s Community-Led Total Sanitation Program that was implemented in 1999, the approach of which was further applied by different stakeholders, in line with support, to the governmental sanitation program (the open-defecation-free campaign) in Nepal [52]. A study completed in Nepal using the NDHS 2011 dataset found that 57% of people had access to improved sanitation; the highest rate was in Kathmandu (100%), and the lowest was in Mahottari (18%) District [47]. The present study found a 27% higher rate of available improved sanitation compared with the previous, similar study in Nepal [36]. This progress has occurred due to open-defecation-free campaigns, community ownership, and collaborative approaches. Every single household in Tehrathum, Lalitpur, Palpa, and Kaski districts had improved sanitation, while, in Sarlahi and Mahottari districts, only 64% of households had improved sanitation [48]. This means the improved sanitation status of plain regions remains challenging. This model could be further expanded in such areas and implemented effectively and sustainably. 

Designating a fixed place for handwashing is considered pivotal in the prevention of communicable diseases and enabling health promotion. The NDHS 2016 data demonstrated ecological disparities in the maintenance of a fixed place for handwashing for households. The present study found that respondents with higher education, respondents living in the hills region, and respondents living in urban areas had a higher prevalence of fixed places for handwashing. Household heads aged 35–44 years were significantly more likely to report having soap and water available in handwashing places compared with other age groups. This might be because men and women aged 35–44 years were more likely to have a higher employment rate (49%) in Nepal [53]. The household wealth index plays a significant role in improving WASH, and employed parents might be able to afford soap and practice handwashing [54].

The Nepal-specific 2019 Multiple Indicator Cluster Survey (MICS) reported that 80% of households had handwashing facilities with soap and water, and the present study’s result was consistent with this rate [55]. This similarity may be due to the MICS and NDHS being conducted in similar regions and geography and with a similar level of knowledge of the participants. The proportional distribution of a fixed place for handwashing in mid–far western hills and mountain regions was comparably lower than in the MICS. It can be assumed that the establishment of a fixed place for handwashing motivates family members to wash their hands because people see the place where they actually can wash hands if soap and water are present. The availability of soap and water at handwashing places was almost the same in Bhutan and Indonesia as in the present study [56]. Ethiopia has very few fixed places for handwashing, but household members wash their hands elsewhere, including in the yard outside. The availability of soap and water separately at the handwashing places was higher than for the 2019 MICS [55]. Handwashing may be less effective if only one of these commodities is present, and, therefore, both soap and water should be available at handwashing places to increase effectiveness.

The lower rates of availability of handwashing facilities may be due to lack of knowledge about the importance of soap, risk perception, high workload, scarcity of water and/or poor economic status [57]. Improved WASH facilities are, therefore, crucial practices for positive health outcomes and the prevention and control of communicable diseases. Construction of toilets, buying soap, and collecting water do not make any sense if the facilities are not used. These measures can only be achieved through service access and education. A review conducted in 2019 found that the poorest households in Nepal had less access to soap and water than the richest households [58]. The current study is similar to that review result. 

This study result is almost similar to a cross-sectional study conducted in 2018 in the Makwanpur district of Nepal after it became ODF, which showed that the availability of improved sanitation at the household level was 92%, access to improved water sources was 90%, and handwashing with soap at critical moments (after defecation or using toilets, after cleaning a child’s bottom or handling nappies, before eating food or feeding a child, before preparing food or handling food, and before breastfeeding) was 43% [58].

The challenges of WASH in Nepal relate to the proper management and sustainability of WASH resources. Based on this study’s findings, in Nepal, there is a geographical discrepancy in WASH, and Madhes Province (which represents the plains region) and Karnali Pradesh (which represents both hills and mountains regions) have poor coverage of sanitation and hygiene. Understanding these geographical inequalities helps to identify the gaps and challenges for both financial and logistics management.

These findings have important policy implications for WASH implementers, researchers, and policymakers. The different rates of WASH coverage by predictor variables are an instrumental tool for future WASH program planning to ensure the equitable and affordable distribution of sustainable WASH practices. This is the right time to explore WASH findings to communicate research-based concerns to authorities and campaign for effective, cost-effective WASH interventions. Based on the findings of this study, the government of Nepal should give WASH priority to Madhes Pradesh and Karnali Pradesh. People who have poor access to WASH services are those with low levels of education, people of low socioeconomic status, and those living in remote geographical areas. The findings suggest that targeted education and services provided are important for improving WASH in Nepal. This can play a significant role in the formulation of context-specific WASH policy at the local, provincial, and country levels. Within this discourse, the local-, provincial-, and central-level governments should focus on timely initiation and sustainable management of WASH services to overcome these challenges and to improve household-level WASH facilities, particularly with a focus on handwashing with soap, in line with the Nepal Health Sector Strategy Implementation Plan and SDG 6 in Nepal. Addressing the WASH component timely is important to support SDG 6 significantly including SDG 1 “no poverty”, SDG 3 “good health and wellbeing”, SDG 4 “quality education”, and SDG 5 “gender equality” [33].

The strength of this study is that it shows a country-specific representation where enough samples were available, thus, allowing for the reflection of current trends of household-level WASH in Nepal. In addition, the study respondents were household heads who are likely to provide accurate data about the household level of WASH since they are well-known persons in the family. A further strength is that the data collectors observed a fixed place for handwashing and the presence of water and soap in the handwashing places, as well as the types of toilets that were available. This is a higher level of evidence than relying on self-report. The prevalence of low rates of improved WASH at the cluster level shows a public health problem in Nepal, and this result may help policymakers to develop WASH plans and programs based on the severity of the problem. Finally, this study covers all the WASH components which were missing in previous studies; for example, Wang et al. in 2019, did not cover handwashing with soap components, and the results related to water and sanitation were underestimated [16]. However, this study has some limitations. Firstly, as the study was cross-sectional, the study results refer to a point in time, meaning it is not possible to determine any causation between explanatory variables and outcomes. Secondly, it was difficult to show results by type of family and “nuclear, joint, and extended or mixed” families may have different prevalence rates and correlations with WASH in Nepal. Thirdly, there was no information on caste or ethnicity in the household survey data. Fourthly, the use of self-reported household improved water source facilities may have led to an overestimation of WASH availability, and this issue, thus, requires further study. This study did not include any possible confounders due to bivariate analysis because researchers intended to look at one-to-one correlation in the selected variables, which might underestimate the results in line with cluster-level WASH distribution. Fifthly, the proportion of women participants was low (31%) in this study, and women are key persons in Nepal who are at home and teach, care for, and support children and other family members in WASH practices. Finally, this study lacked a supply-side factor of governments, ministries, and organizations, as well as political factors that need to be considered in future studies.

## 5. Conclusions

The rates for access to an improved water source and improved sanitation are higher than those for the availability of soap and water in handwashing places. Education and geographical disparities are the key factors associated with having reduced access to WASH in Nepal. These findings suggest the need to focus on health promotion, advocacy, WASH services, and policy and strategy approaches for household-level WASH. A collaborative intervention beyond health, such as agriculture, education etc., must be endorsed for total WASH services at the household level in Nepal. This study provides a blueprint to estimate the prevalence and correlates of WASH and spatial distribution of unimproved WASH, and it incorporates forward-thinking development approaches to WASH in Nepal. Further study is suggested to explore household-level factors that might contribute to higher provision and uptake of WASH, especially handwashing with soap and water. A longitudinal study regarding the exposure variables and WASH would be necessary to assess the causal nature of any relationships.

## Figures and Tables

**Table 1:** Predictor variables included in the study.

Variables	Class
Age of the household head (in years)	15–24; 25–34; 35–44; 45 and above
Sex of the household head	Male; Female
Education level	No education: unable to read or write; Primary: completed Year 5 schooling; Secondary: completed Year 8 schooling; School Leaving Certificate (SLC) or higher: completed Year 10 or above schooling; Do Not Know
Marital status	Married; Unmarried; Widowed/divorced
Number of household family members	1–2; 3–4; 5–6; 7+
Place of residence	Rural; Urban
Ecological zone	Plains; Hills; Mountains
Province	1, not named yet ^1^; 2, Madhes Pradesh; 3, Bagmati Pradesh; 4, Gandaki Pradesh 5, Lumbini Pradesh; 6, Karnali Pradesh; 7, Sudurpashchim Pradesh
Household wealth index	Poor; Middle; Rich
Distance to a water source	≤30 min walk: 30 min or less walking time to water source; >30 min walk: more than 30 min walking time to water source; Do Not Know

^1^ The new constitution of Nepal, adopted on 20 September 2015, divided the country into 7 federal provinces by splitting (Nawalparasi and Rukum) and grouping the existing districts. Each of their local governments was given the right to choose a name. While all other provinces have decided their names, Province 1 still has to reach a consensus on the name.

**Table 2:** Socio-demographic and WASH characteristics of respondents (*n* = 11,040).

Variables	Class	Weighted Frequencies (*n*)	Weighted Percentages (%)
Age of household head (in years)	15–24	625	5.7
	25–34	2240	20.3
	35–44	2562	23.2
	45 and above	5613	50.8
Sex of household head	Male	7581	68.7
	Female	3459	31.3
Education of household head	No education	4310	39.1
	Primary	2492	22.5
	Secondary	2947	26.7
	Higher	1272	11.5
	Do not know	19	0.2
Marital status of household head	Married	9499	86.0
	Unmarried	337	3.1
	Widowed and divorced	1204	10.9
Number of family members	1–2	2160	19.6
	3–4	4146	37.6
	5–6	3048	27.6
	7+	1685	15.2
Place of residence	Rural	6019	54.5
	Urban	5021	45.5
Ecological zone	Plains	5125	46.4
	Hills	5134	46.5
	Mountains	781	7.1
Province	1 (not named yet)	2004	18.2
	2 (Madhes)	2014	18.2
	3 (Bagmati)	2521	22.9
	4 (Gandaki)	1173	10.6
	5 (Lumbini)	1793	16.2
	6 (Karnali)	619	5.6
	7 (Sudurpashchim)	916	8.3
Wealth index of household	Poor	4459	40.4
	Middle	2065	18.7
	Rich	4516	40.9
Source of water	Improved	10,543	95.5
	Unimproved	497	4.5
Distance to a water source	≤30 min walk	10,476	94.9
	>30 min walk	562	5.1
	Do not know	2	0.1
Type of sanitation	Improved	9246	83.8
	Unimproved	1794	16.2
Handwashing place	Fixed	8936	80.9
	Non-fixed	2075	18.8
	Missing (not observed)	29	0.3
Soap and water	Available	5185	46.9
	Not available	5827	52.8
	Missing (not observed)	28	0.3

**Table 3:** Bivariate analysis of correlates of having access to improved water sources, improved sanitation, availability of soap and water, and access to a fixed place for handwashing.

Variables	Class	Improved Water	Improved Sanitation	Soap and Water	Fixed Place
		OR (95% CI)	OR (95% CI)	OR (95% CI)	OR (95% CI)
Age of household head (in years)	15–24	1.14 (0.73–1.78)	1.02 (0.76–1.39)	1.11 (0.87–1.44)	1.02 (0.81–1.27)
	25–34	0.95 (0.70–1.27)	0.92 (0.78–1.07)	1.13 (0.99–1.28)	1.13 (0.96–1.32)
	35–44	1.04 (0.84–1.29)	1.07 (0.91–1.26)	1.16 (1.04–1.29)	1.05 (0.92–1.22)
	45 and above	1	1	1	1
Sex of household head	Male	1	1	1	1
	Female	0.87 (0.72–1.06)	1.02 (0.85–1.22)	0.96 (0.86–1.07)	0.95 (0.83–1.09)
Education of household head	No education	1	1	1	1
	Primary	1.11 (0.90–1.38)	1.95 (1.63–2.33)	1.78 (1.58–2.02)	1.62 (1.39–1.89)
	Secondary	1.81 (1.28–2.57)	3.37 (2.71–4.19)	2.85 (2.49–3.26)	2.41 (1.99–2.92)
	Higher	3.51 (1.86–6.62)	13.43 (7.91–22.86)	7.09 (5.73–8.70)	6.01 (4.47–8.07)
Marital status of household head	Married	1	1	1	1
	Unmarried	2.59 (0.94–7.16)	2.50 (2.36–8.56)	2.25 (1.66–3.31)	1.96 (1.32–2.90)
	Widowed and divorced	0.92 (0.68–1.23)	0.95 (0.73–1.15)	0.79 (0.69–0.92)	0.76 (0.62–0.94)
Number of family members	1–2	0.96 (0.74–1.24)	0.96 (0.81–1.14)	0.80 (0.69–0.92)	0.83 (0.70–0.99)
	3–4	1	1	1	1
	5–6	0.83 (0.65–1.05)	0.77 (0.65–0.91)	0.77 (0.69–0.86)	0.78 (0.68–0.88)
	7+	1.37 (0.98–1.92)	0.56 (0.0.45–0.70)	0.71 (0.60–84)	0.78 (0.65–0.93)
Place of residence	Rural	1	1	1	1
	Urban	1.10 (0.63–1.93)	2.30 (1.52–3.49)	3.58 (2.78–4.64)	2.36 (1.82–3.06)
Ecological zone	Plains	1	1	1	1
	Hills	0.32 (0.16–0.64)	6.41 (4.39–9.37)	1.25 (0.95–1.64)	1.33 (1.01–1.77)
	Mountains	0.62 (0.26–1.51)	4.30 (2.52–7.35)	0.48 (0.31–0.75)	0.61 (0.41–0.91)
Province	1 (not named yet)	1	1	1	1
	2 (Madhes)	1.01 (0.30–3.42)	0.15 (0.08–0.28)	0.54 (0.37–0.79)	0.44 (0.29–0.67)
	3 (Bagmati)	0.54 (0.21–1.36)	1.82 (0.93–3.54)	1.90 (1.26–2.86)	1.36 (0.87–2.11)
	4 (Gandaki)	0.54 (0.24–1.20)	2.92 (1.52–5.61)	1.23 (0.84–1.80)	2.28 (1.50–3.46)
	5 (Lumbini)	0.99 (0.41–2.41)	1.06 (0.51–2.20)	0.80 (0.54–1.18)	1.06 (0.68–1.66)
	6 (Karnali)	0.19 (0.09–0.43)	2.69 (1.52–4.76)	0.38 (0.25–0.58)	0.49 (0.31–0.77)
	7 (Sudurpashchim)	0.65 (0.28–1.48)	1.93 (1.03–3.58)	0.96 (0.64–1.45)	0.99 (0.61–1.63)
Wealth index of household	Poor	0.22 (0.10–0.50)	0.17 (0.13–0.24)	0.13 (0.10–0.15)	0.25 (0.20–0.31)
	Middle	0.72 (0.31–1.66)	0.13 (0.10–0.18)	0.22 (0.18–0.26)	0.33 (0.26–0.42)
	Rich	1	1	1	1
Distance to a water source	>30 min walk	0.07 (0.04–0.12)	1.03 (0.69–1.54)	0.36 (0.25–0.52)	0.39 (0.27–0.57)
	≤30 min walk	1	1	1	1

## Data Availability

The data used in the study are publicly available from the DHS program website (https://dhsprogram.com/data/available-datasets.cfm, accessed on 12 November 2018).
